# Food cravings after bariatric surgery: comparing laparoscopic sleeve gastrectomy and Roux-en-Y gastric bypass

**DOI:** 10.1007/s40519-023-01636-2

**Published:** 2024-01-12

**Authors:** Afton M. Koball, Gretchen E. Ames, Alec J. Fitzsimmons, Kara J. Kallies, Barb A. Bennie

**Affiliations:** 1 Department of Psychiatry and Psychology, Mayo Clinic, Rochester, MN USA; 2 Department of Psychiatry and Psychology, Mayo Clinic, Jacksonville, FL USA; 3 Department of Medical Research, Gundersen Health System, La Crosse, WI USA; 4 Public & Community Health, Medical College of Wisconsin, Milwaukee, WI USA

**Keywords:** Sleeve gastrectomy, Roux-en-Y gastric bypass, Food cravings, Food craving inventory, Patient health questionnaire-9, Generalized anxiety disorders-7, Modified Yale Food Addiction Scale, Binge eating disorder

## Abstract

**Background:**

Research suggests that food choices, preferences, and tastes change after bariatric surgery, but evidence regarding changes in food cravings is mixed.

**Objectives:**

The primary aim of this cohort study was to compare food cravings during the first year following bariatric surgery in patients who had undergone sleeve gastrectomy (SG) versus Roux-en-Y gastric bypass (RYGB).

**Setting:**

Integrated multispecialty health system, United States.

**Methods:**

Patients aged ≥ 18 years seen between May 2017 and July 2019, provided informed consent, completed the Food Craving Inventory (FCI), and had ≥ 1 year of follow-up after undergoing primary SG or RYGB were included in the study. Secondary data captured included psychological and behavioral measures. Preoperative and postoperative (3, 6, 9, and 12 months) FCI scores of patients who underwent SG and RYGB were compared.

**Results:**

Some attrition occurred postoperatively (*N* = 187 at baseline, 141 at 3 months, 108 at 6 months, 89 at 9 months, and 84 at 12 months). No significant relationship between pre- or postoperative food cravings and surgery type was found except on the carbohydrate subscale. Patients with higher preoperative food addiction symptoms were not more likely to experience an earlier reoccurrence of food cravings during the first 12 months after surgery. Likewise, patients with higher levels of preoperative depression and anxiety were not more likely to have early reoccurrence of food cravings during the first 12 months after surgery; however, those with higher PHQ9 scores at baseline had uniformly higher food craving scores at all timepoints (pre-surgery, 3 m, 6 m, 9 m, and 12 m).

**Conclusions:**

Results suggest that food cravings in the year after bariatric surgery are equivalent by surgery type and do not appear to be related to preoperative psychological factors or eating behaviors.

**Level of evidence:**

Level III: Evidence obtained from well-designed cohort.

**Supplementary Information:**

The online version contains supplementary material available at 10.1007/s40519-023-01636-2.

## Highlights

- Food cravings are significantly reduced after bariatric surgery, although they generally do not differ by surgery type. (With the exception of carb cravings, specifically, which were higher for those undergoing RYGB than SG).
- Patients with higher levels of preoperative depression and anxiety were not more likely to have early reoccurrence of food cravings during the first 12 months after surgery; however, those with higher PHQ9 scores at baseline had uniformly higher food craving scores at all timepoints (pre-surgery, 3 m, 6 m, 9 m, and 12 m).
- Patients with higher preoperative food addiction symptoms were not more likely to experience an earlier reoccurrence of food cravings during the first 12 months after surgery.

**Supplementary Information:**

The online version contains supplementary material available at 10.1007/s40519-023-01636-2.

## Introduction

Bariatric surgery is considered the most effective intervention for obesity and many of its medical comorbidities and should be considered for individuals with metabolic disease and Body Mass Index (BMI) of 30–34.9 kg/m^2^ or for individuals with body mass index (BMI) ≥ 35 kg/m^2^ regardless of comorbidity status [1–3]. Some of the metabolic mechanisms of action that are believed to contribute to the effectiveness of bariatric surgery are changes in hunger and satiety gut hormones, bile acid signaling, gut microbiota, inulin sensitivity, and neural pathways that regulate appetite and fat storage [4]. These changes affect patients’ food consumption via altered food choices, preferences, and tastes [5–9] and are important to how individuals achieve and sustain significant weight loss following surgery.

Alterations in one’s relationship with food (e.g., reasons for eating) and eating habits (e.g., unplanned snacking) from pre- to post-surgery are important components of success. After surgery many people prefer to eat smaller, more frequent meals that are less calorically dense, less sweet, and lower in fat [7, 10–13]. Protein intake generally increases in the first postoperative year as fat intake is reduced, and carbohydrate intake remains unchanged [6]. Physical effects of eating certain foods also can affect food choice and preference. For example, dumping syndrome occurs when high-sugar foods are consumed, and patients may avoid eating these foods to ward off unpleasant side effects [7]. Although physiological factors explain some of the changes in food preferences, psychological (e.g., emotion regulation, depression) and social (e.g., social pressure) factors may also play a role [14]. Changes in food preferences following surgery have been found to last up to 5 years postoperatively, although there is evidence that these preferences slowly return to the preoperative preference state [15].

Another important contributing factor in food consumption after bariatric surgery is food cravings, which have been defined as an “intense desire to consume a particular food or food type that is difficult to resist” [16]. Previous research has suggested that food cravings change after bariatric surgery and may affect weight loss, yet the literature remains mixed [17–20].

Leahey and colleagues [17] compared the food cravings of patients who had undergone Roux-en-Y gastric bypass (RYGB) with those of patients in a weight control group who did not undergo surgery. They found a significant decrease in food cravings and consumption post-bariatric surgery, with the most significant reduction happening within the first 3 months. By 6 months after surgery, cravings and consumption gradually increased, although they remained significantly lower than before surgery. Sudan et al. [18] compared the food cravings of patients who had RYGB with those of a control group who underwent cholecystectomy and found that although consumption of craved foods decreased significantly in the RYGB group compared with the control, there was no change in food cravings between groups within the first 12 months after surgery. When examining the relationship between food cravings and postoperative weight loss, the literature is similarly mixed, with some finding no relationship [17, 18], some finding a negative relationship (higher cravings = lower excess weight loss) [20], and some finding a positive relationship (higher cravings = higher weight loss) [19].

While not all food craving is pathological, some literature has identified it’s overlap with addictive eating behaviors (i.e., food addiction). Measures of food addiction (e.g., the Yale Food Addiction Scale; YFAS) commonly include items related to craving [21]. Previous literature has suggested that individuals with food addiction more frequently endorse food craving factors, including intention to consume food, relief from negative affect after eating, loss of control overeating, preoccupation with food, hunger, etc. [22, 23]. In addition, food addiction symptoms may be predicted by food cravings [24]. A recent meta-analysis of food addiction and bariatric surgery found that pre-operative food addiction was related to psychological and behavioral factors, including food cravings [25].

Although some research has examined differences in food preferences, choices, and taste by surgery type [8, 9], no research has specifically examined differences in food craving changes pre- to post-surgery between patients who underwent RYGB and sleeve gastrectomy (SG). While one recent meta-analysis of food preference changes after bariatric surgery found that most studies utilized samples of patients who underwent RYGB rather than SG, only one study involved both surgery types [15]. Further research on differences in food cravings by surgery type is warranted given that, compared with RYGB, SG may allow for better tolerance of foods post-operatively, may have unique hormone-modifying implications, and may result in different patterns of dietary intake and eating habits post-operatively [26]. While long term studies of SG are less common than RYGB, it has been suggested that individuals undergoing SG are at higher risk of long-term weight recurrence [27] and behavioral factors involving food cravings (e.g., graze eating, loss of control eating) may be one reason for this finding.

The primary aim of this study was to examine differences in cravings between individuals who undergo SG versus RYGB during the first year following bariatric surgery. Given the long-term durability and greater expected weight loss achieved with RYGB compared with SG [28], we hypothesized that patients who have undergone SG might experience higher levels of food cravings post-surgery and/or earlier reoccurrence of these cravings.

In addition, research has consistently demonstrated that while many maladaptive eating behaviors often remit for some time after surgery, they may reoccur up to several years after surgery [29, 30]. Consistent with literature outlined above**,** we hypothesized that those with maladaptive eating behaviors (e.g., food addiction and/or craving) before surgery would be more likely to have earlier recurrence of food cravings after surgery than those who did not. Recurrence of maladaptive eating behaviors is often associated with higher psychological distress and impairment, such as depression and anxiety [31]. Therefore, we hypothesized that patients with higher baseline levels of depression and anxiety symptoms would also have higher levels of an earlier recurrence of food cravings after surgery than would patients who reported minimal mood symptoms at baseline.

## Methods

### Study design

The current study examined food cravings before and after bariatric surgery at routine medical visits with a Metabolic and Bariatric Surgery Accreditation and Quality Improvement Program (MBSAQIP)-accredited bariatric program at a medium-sized Midwestern hospital. Patients were offered participation in this study at their first postoperative visit with a registered dietitian and, if they were interested, gave informed consent to be included in the study. Questionnaires were given to patients and completed at their pre-operative visit as well as at 3-, 6-, 9-, and 12-month post-operative visits at time of check in to their clinic appointment. Patients were informed that they could elect not to complete the study measures or could withdraw from the study at any time without any impact on their medical care. Patients who had surgery between May 2017 to July 2019 and who had at least 1 year of postoperative follow-up were included in analyses. Data collection was stopped prematurely due to the COVID-19 pandemic. All patients were 18 years of age or older and had either primary RYGB or SG. Patients were excluded if they were undergoing a revisional procedure or if they did not complete the food cravings measure at baseline. An assessment battery was conducted at a preoperative visit (pre-surgical psychological evaluation, baseline), which included a measure of food cravings and other relevant psychosocial assessments used in routine clinical care. Patients were given follow-up assessments of food cravings at 3, 6, 9, and 12 months postoperatively when they met with a registered dietitian. A review of patients’ electronic health records was conducted to capture demographic variables, and surgery type. This study was approved by the Institutional Review Board at the study hospital.

### Measures

The primary measure of interest, the Food Craving Inventory (FCI) [16], was used to examine both food cravings and consumption of craved foods. Respondents were asked to indicate how often they have craved each of 28 food items over the past 30 days on a 5-point Likert scale from 1 (never) to 5 (always). In addition, participants were asked how often they ate a particular food that they may have craved. An overall food craving score was calculated (mean of all 28 items; higher scores indicate higher food cravings), as well as scores for 4 subscales representing mean ratings for high-sugar foods, fast foods, high-fat foods, and high-carbohydrate foods.

Measures of mood included the Patient Health Questionnaire-9 (PHQ-9) and the Generalized Anxiety Disorders-7 (GAD) questionnaire. The PHQ-9 is a 9-item self-report measure of depression symptoms with strong internal consistency and test–retest reliability [30]. Patients answer items on a scale of 0 (not at all) to 3 (nearly every day). The total PHQ-9 score was computed by summing responses to the 9 items [21]. The GAD-7 is a 7-item self-report measure designed to identify probable cases of GAD with a cut point of 10 or greater. Patients answer items on a scale of 0 (not at all) to 3 (nearly every day) [31]. The total GAD-7 score was computed by summing responses.

The Yale Food Addiction Scale (m-YFAS) [21, 32, 33] was used to measure food addiction symptoms and is a 13-item self-report measure that examines the severity and clinical significance of food addiction based on DSM-5 criteria for substance abuse and dependence. Patients answered items on a scale ranging from 0 (never) to 7 (every day). Severity of food addiction was categorized as mild (symptom count = 2 or 3 plus clinical significance ≥ 1 [items 5 and 6, which measure distress and significant life problems related to eating behaviors]), moderate (symptom count = 4 or 5 plus clinical significance ≥ 1), or *severe* (symptom count ≥ 6 plus clinical significance ≥ 1). Responses to item #10 on the m-YFAS 2.0 (“I had such strong urges to eat certain foods that I couldn’t think of anything else”) were analyzed to compare pre- and postoperative food cravings. A small number of patients were administered the full YFAS 2.0 instrument [34]. In these cases, only the subset of questions that comprise the m-YFAS 2.0 were extracted, and patients were then scored according to the m-YFAS 2.0 protocol.

### Statistical methods

The reliability of the FCI instrument was assessed using Cronbach’s alpha on the total score along with all subscale scores at each study timepoint. Pearson’s correlation coefficients between the four sub-scale measurements were computed at each timepoint. These measures were used to benchmark the reliability and internal consistency observed in our study relative to White et al. [16].

To examine differences in cravings between individuals who undergo SG versus RYGB during the first year following bariatric surgery, we fit a linear mixed effect model using the total FCI score as the response variable. Timepoints with four discrete values (pre-surgery, 3 months, 6 months, 9 months, and 1-year post-surgery) was treated as a within subject fixed effect, while surgery type was treated as a between subject fixed effect. The interaction of time and surgery type was included in the model to allow for differing patterns in FCI over time for the two surgery types. Patients were treated as random subjects in the model. Post-hoc pairwise comparisons were carried out for significant model terms using a false discovery rate (FDR) adjustment for multiple comparisons. This same linear mixed effect model structure was applied with each of the FCI subscale scores serving as the response variable: sweets, fast food fats, high fats, and carbs.

To relate maladaptive eating behaviors along with levels of anxiety and depression before surgery to food craving patterns, we again used linear mixed models. For this set of models, the total FCI score served as the response variable. Each of the pre-metrics of food addiction, anxiety, or depression was used an independent variable, while timepoint served as the within subject fixed effect. The pre-metrics of food addiction, anxiety, or depression were used one-at-a-time in these models due to high-collinearity. This allowed us to assess the importance of each pre-metric separately in predicting FCI scores. One linear mixed effect model was fit for each of these pre-metrics: mYFAS 2.0 symptom count, mYFAS 2.0 food addiction diagnosis (yes/no), mYFAS 2.0 craving symptom (yes/no), PHQ-9 score, and GAD-7 score.

Bivariate correlations between percent excess weight loss and FCI total scores at various timepoints were used to assess connections between food craving and weight loss. Descriptive statistics are reported as means and standard deviations or counts and percentages. All analysis was completed using R version 4.2.3 with the lme4, grafify, sjPlot, gtsummary, and tidyverse packages. The threshold for statistical significance was 0.05 for all hypothesis tests.

## Results

Adults who underwent primary bariatric surgery (RYGB or SG) and completed the FCI in its entirety at baseline, were included in the study (*N* = 187). Average patient age was 46 ± 12 years, most patients were women (158/187 = 84%), and 49.7% underwent SG (93/187). Some attrition occurred postoperatively (*N* = 187 at baseline, 141 at 3 months, 108 at 6 months, 89 at 9 months, and 84 at 12 months). Internal consistency for the overall FCI tool and its subscales was acceptable (Cronbach’s alpha > 0.70) at all timepoints, except for the fast-food subscale at all timepoints and the sweets subscale at 9-month post-surgery.

### Comparing food cravings by surgery types

**Fig. 1:**
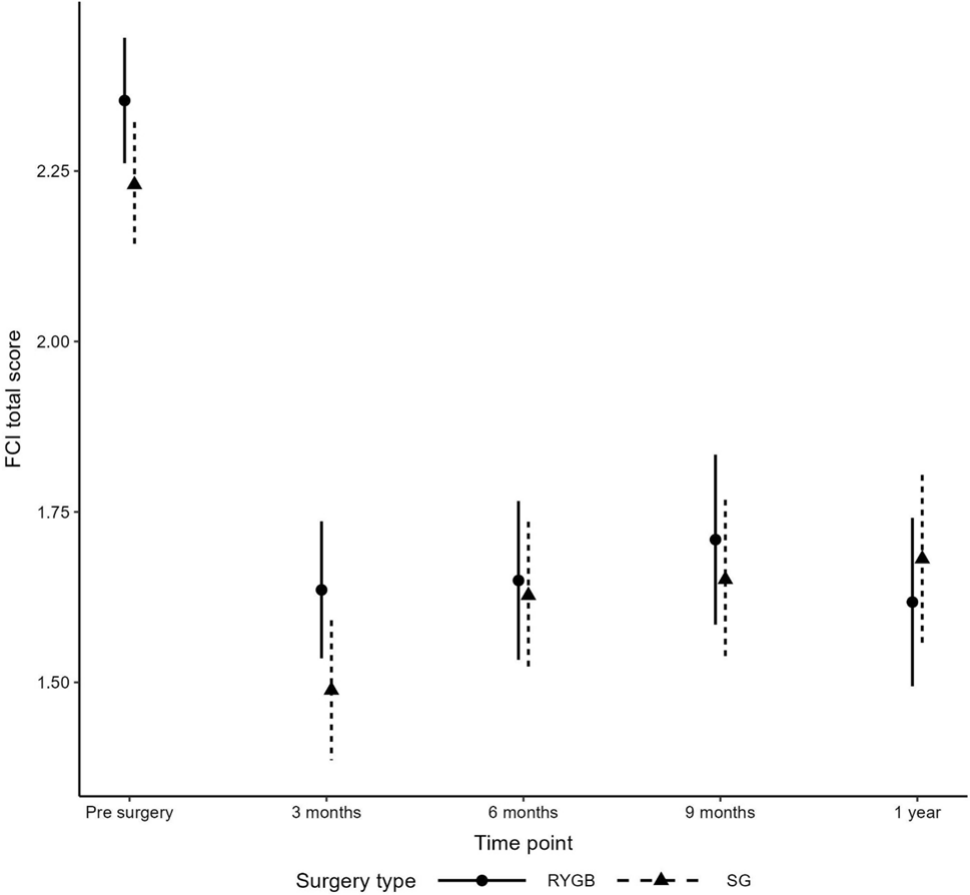
Mean total food craving inventory scores by surgery type at study timepoints (preoperative, 3, 6, 9, and 12 months)

For the total FCI score, there were not significant differences between surgery types (interaction *P* = 0.16 and surgery type main effect *P* = 0.30). There were significant differences in food cravings over time (*P* < 0.001) with total FCI score decreasing at all pre- to post-surgery timepoints (3 m, 6 m, 9 m, and 1y; *P* < 0.001). FCI total score at 3-month post-surgery was significantly lower than at 9-month post-surgery (*P* = 0.02), see Fig. 1.

**Fig. 2:**
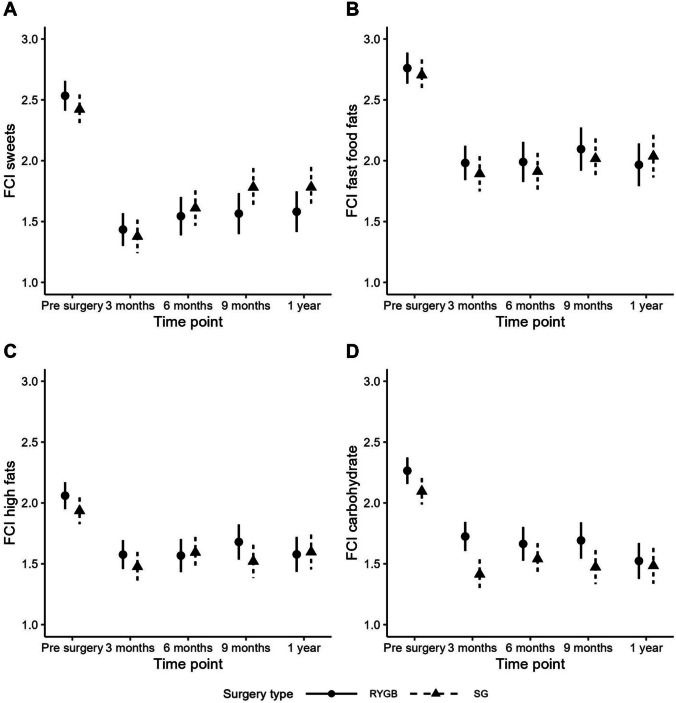
Mean subscale food craving inventory scores by surgery type at study timepoints (preoperative, 3, 6, 9, and 12 months). **A** High-sugar, **B** fast food, **C** high-fat, and **D** high-carbohydrate

When modeling the FCI subscales, only the sweets and carbs subscales showed significant differences between surgery types, see Fig. 2. For the sweet’s subscale, we found a significant difference in the pattern of FCI scores over time between SG and RYGB surgeries (interaction *P* = 0.02). However, in post-hoc testing on the sweets sub-scale, the difference between surgery types was not statistically significant at any timepoint (lowest *P* = 0.07 and 0.09 at time 9 m and 1y, respectively). When averaging across surgery types there are significant differences over time in measured sweets cravings; in particular, the sweets FCI score decreased pre- to post-surgery at all timepoints (3 m, 6 m, 9 m, and 1y; *P* < 0.0001) and the sweets FCI score at 3-month post-surgery was significantly lower than at all later timepoints (6 m, 9 m, and 1y; *P* = 0.007, 0.0001, 0.0001, respectively). For the carbohydrates sub-scale score, there was a significant difference between surgery types (surgery type main effect *P* = 0.01). The interaction *p* value was not statistically significant (interaction *P* = 0.15) suggesting that the difference in mean carb sub-scores between surgery types was similar for all study timepoints with RGYB having higher mean carb scores than SG surgeries (estimated difference = 0.17, SE = 0.6). When averaging across surgery types there are significant differences in the carbs sub-scale over time; in particular, the carb FCI score decreased pre- to post-surgery at all timepoints (3 m, 6 m, 9 m, and 1y; *P* < 0.0001). For both the fast-food fats and high fats FCI sub-scale scores, pre-surgery means are significantly higher than all post-surgery timepoints (3 m, 6 m, 9 m, and 1y; *P* < 0.0001) (Table 1).

**Table 1:** Bariatric surgery patients’ preoperative demographic, anthropometric, and psychological and behavioral measure values

Characteristic	Value
Sex (*N* = 187), *n* (%)	
Female	158 (84)
Male	29 (16)
Age, mean ± SD, years	46 ± 12
Procedure (*N* = 204), *n* (%)	
Sleeve gastrectomy	93 (50)
Roux-en-Y gastric bypass	94 *n*
Psychological and behavioral measures	
PHQ-9, mean score ± SD (*N* = 164)	4.7 ± 4.2
GAD-7, mean score ± SD (*N* = 168)	2.9 ± 3.5
m-YFAS Score (*N* = 131)	
No food addiction	120 (91.6)
Food addiction	11 (8.4)

*SD* standard deviation, *FCI* food craving inventory, *PHQ-9* Patient Health Questionnaire-9, *GAD-7* General Anxiety Disorder-7, *m-YFAS* Modified Yale Food Addiction Scale, *QEWP-5* Questionnaire of Eating and Weight Patterns-5

### Food cravings and maladaptive eating behaviors, anxiety, or depression before surgery

There was a significant association between mYFAS 2.0 symptom count and the pattern of total FCI scores over time (interaction *P* < 0.001). In particular, prior to surgery we estimate the mean FCI score is 0.11 (SE = 0.02) units higher for each additional mYFAS 2.0 symptom reported. This gap is estimated to drop after surgery. In particular, the gap in FCI score per each additional mYFAS 2.0 symptom reported pre-surgery is estimated to be 0.01 at 3 months, 0.03 at 6 months, 0.04 at 9 months, and 0.03 at 12 months (Table 2).

**Table 2:** Change in food craving inventory (FCI) scores from baseline to 3-, 6-, 9-, and 12-month post-surgery

	Contrast	Estimate	SE	*p* value
Overall FCI score				
	Pre-surgery to 3 m	0.730	0.037	< .0001
	Pre-surgery to 6 m	0.653	0.041	< .0001
	Pre-surgery to 9 m	0.612	0.044	< .0001
	Pre-surgery to 12 m	0.642	0.045	< .0001
High sugar FCI score				
	Pre-surgery to 3 m	1.072	0.052	< .0001
	Pre-surgery to 6 m	0.901	0.057	< .0001
	Pre-surgery to 9 m	0.805	0.061	< .0001
	Pre-surgery to 12 m	0.796	0.062	< .0001
Fast food FCI score				
	Pre-surgery to 3 m	1.072	0.052	< .0001
	Pre-surgery to 6 m	0.901	0.057	< .0001
	Pre-surgery to 9 m	0.805	0.061	< .0001
	Pre-surgery to 12 m	0.796	0.062	< .0001
High-fact FCI score				
	Pre-surgery to 3 m	0.472	0.041	< .0001
	Pre-surgery to 6 m	0.417	0.045	< .0001
	Pre-surgery to 9 m	0.398	0.049	< .0001
	Pre-surgery to 12 m	0.410	0.050	< .0001
High carbohydrate FCI score				
	Pre-surgery to 3 m	0.611	0.045	< .0001
	Pre-surgery to 6 m	0.578	0.050	< .0001
	Pre-surgery to 9 m	0.598	0.053	< .0001
	Pre-surgery to 12 m	0.677	0.054	< .0001

*SE*, standard error, *FCI* food craving inventory

Estimated differences-based linear mixed models are average over levels of surgery type

*p* values are adjusted to control false discover rate across contrasts

There was a significant association between mYFAS 2.0 food addiction “diagnosis” and the pattern of total FCI score over time (interaction *P* = 0.04). Pre-surgery we expect the mean FCI score to be significantly higher for those with a “yes” measure of food addiction compared to those with “no” food addiction (estimated difference = 0.48, SE = 0.14, *P* = 0.001). This gap decreases to be non-significant for all post-surgery timepoints (3 m *P* = 0.91; 6 m *P* = 0.52, 9 m *P* = 0.38, 1y *P* = 0.61), see Table 3. There was not a significant association between mYFAS 2.0 item 10 response and the pattern of total FCI score over time (interaction *P* = 0.37 and main effect *P* = 0.74).

**Table 3:** Comparison of pre-surgery, 3 m, 6 m, 9 m and 12 m food craving inventory (FCI) scores between patients who meet mYFAS criteria and those who did not

Timepoint	Contrast	Estimate	SE	*p* value
Pre-surgery	No–Yes	− 0.478	0.144	0.001
3 months	No–Yes	0.019	0.169	0.910
6 months	No–Yes	− 0.110	0.170	0.518
9 months	No–Yes	− 0.242	0.276	0.382
12 months	No–Yes	− 0.145	0.281	0.605

*SE* standard error, *FCI* food craving inventory, *m-YFAS* Modified Yale Food Addiction Scale

Estimates of difference in mean food craving score-based linear mixed model between those without and with mYFAS 2.0 food addiction “diagnosis” (Yes/No) at each study timepoint

*p* values are adjusted to control false discovery rate across contrasts

Similarly, there was not a significant association between GAD-7 total score and the pattern of total FCI score over time (interaction *P* = 0.90 and main effect *P* = 0.87). For the total PHQ-9 score, there is not a significant association between the score and the pattern of total FCI score over time (interaction *P* = 0.92). However, there was an additive shift in FCI scores that applies uniformly across all timepoints (main effect *P* = 0.04). There was an estimated increase in average total FCI score by approximately 0.015 (SE = 0.007) units for each additional point on the PHQ-9 assessment.

### Food cravings and weight loss

No significant correlations were noted between FCI scores and percent excess weight loss at any timepoints.

## Discussion

The literature on food cravings and bariatric surgery is mixed [17–20]. To our knowledge, this is the first study to specifically explore the relationship between food cravings and type of bariatric surgery (SG versus RYGB). Results from this study were contrary to what was hypothesized with no significant relationship between food cravings (pre- and post-op) and surgery type emerging. Higher food addiction symptoms and “diagnosis” was associated with greater experiences of food cravings before surgery but not after. No significant relationship emerged between anxiety symptoms preoperatively and food cravings postoperatively, although there was an increase in food cravings for those with higher depression symptoms.

While there were not differences in food cravings between surgery types, food cravings did significantly decrease overall (across both surgery types) pre- to post-op at all timepoints, with the 3-month postoperative period being the lowest point in reported food cravings. Over time, food cravings appear to gradually increase, although remain lower than pre-op levels. Cravings for sweet foods decreased for both surgery types and, similar to the overall food cravings score, were lowest at 3-month post-op. Carbohydrate cravings were higher for patients who underwent RYGB at all post-operative timepoints, although all patients, regardless of surgery type, experienced a reduction in carb cravings after surgery. Cravings for fast-food fats and high fats similarly decreased pre- to post-surgery.

These results mirror previous research on physiological changes that occur in the early post-operative period. These changes, understandably impact a patient’s subjective experience of food cravings due to changes in hepatic insulin resistance leading to lowered basal glucose concentrations, rapid digestion and absorption of nutrients, and modification of gut hormone release and regulation within the first few days following surgery [34]. Clearly, these physiological mechanisms strongly impact patient-reported cravings early on (within 3 months) after surgery. It should be noted that less is known about food preferences, tastes, and cravings postoperatively in patients who underwent SG, because much of the research has been conducted in patients who underwent RYGB. Furthermore, emerging data suggest that long-term weight recurrence after SG is greater than that after RYGB [35]; it remains to be seen how food craving changes may or may not play a role in long-term weight recurrence.

When examining the association between food addiction and cravings, study results suggested that pre-surgery, patients with higher food addiction symptoms have higher food cravings. After surgery, however, this association becomes nominal. Similarly, individuals with a “diagnosis” of food addiction reported higher food cravings pre-surgery but not post-operatively. Both surgery types appear to ameliorate the impact of food addiction and cravings. It has recently been suggested that bariatric surgery could serve as a “treatment” for food addiction with a small body of literature suggesting that food addiction symptoms decrease within the first year after surgery [25, 36–38]. Results from the current study are in line with this literature and further highlight the benefits of bariatric surgery on maladaptive eating behaviors in the early post-operative period. Longer term follow-up on the potential return of food cravings and how this relates to food addiction and other maladaptive eating behaviors given the impact on weight recurrence potential [39]. Notably, and related to the current study, less is known about food preferences, tastes, and cravings postoperatively in patients who underwent SG, because much of the research has been conducted in patients who underwent RYGB. Furthermore, emerging data suggest that long-term weight recurrence after SG is greater than that after RYGB [35]. Evaluating these constructs by surgery type may be important over the long term.

Contrary to original hypotheses, no relationship between food cravings and anxiety emerged. However, individuals with higher depression scores did appear to have higher food cravings at all timepoint pre- and post-surgery. It has been suggested that both anxiety and depression commonly decrease after surgery [40]. Understanding pre-operative behavioral and psychological symptoms are important in predicting post-operative outcomes; the constructs of demoralization and negative emotions (which align with depression) prior to surgery predict eating behaviors and quality of life post-operatively [41]

### Strength and limits

Strengths of this study include being the first study to explore the relationship between food cravings and type of bariatric surgery. The longitudinal study design permitted statistical exploration and inference that are not commonly seen by cross-sectional studies.

Some limitations should be noted in this study, which included a relatively homogenous sample of patients from a single bariatric surgery center. This study included only patients who received primary bariatric surgery; follow-up study in patients who underwent revisional surgery may provide important information on the interplay between food cravings and weight recurrence (assuming that was the reason for a revisional surgery) postoperatively. As with many studies post-bariatric surgery, this study experienced some attrition of patients after surgery; perhaps the patients who kept their follow-up appointments and completed postoperative questionnaires were more motivated, had better outcomes, and were more engaged with their bariatric team than those who did not. Further study on patients struggling to attend postoperative appointments and/or who have lower motivation would be interesting. Finally, the short postoperative period included in this study may have led to fewer experiences of postoperative cravings. Many patients who are several years out from surgery begin to experience the return of food cravings; future long-term study of postoperative food cravings is warranted (Additional file 1: Table S1).

### What is already known on this subject?

Alterations in one’s relationship with food and eating impacts pre- and post-operative outcomes, although, prior to this study, had not been explored between surgery types. Both physiological factors and psychological factors can impact the presentation of food addiction [7, 14]. Previous research has found that food cravings change after bariatric surgery and may affect weight loss, yet the literature remains mixed [17–20]. Food addiction has previously been identified as one factor that is related to food cravings, and which may have implications for bariatric surgery [25]. Similarly, mood impairment has been associated with maladaptive eating behaviors and may relate to food cravings [31].

### What this study adds?

Findings from this study add to the existing literature that bariatric surgery is an effective treatment for food cravings and other problematic eating behaviors, at least in the short term. Both SG and RYGB lead to significant and immediate reduction in food cravings postoperatively; data from this study suggest that neither surgery type seems more or less likely to result in food cravings during the first year after surgery. Patients with preoperative food addiction symptoms experience higher food cravings, which is ameliorated after surgery. Anxiety did not seem to affect food cravings in the first postoperative year, although depression and food cravings were associated. It appears that both the SG and RYGB are helpful options for reducing food cravings which can contribute to substantial weight loss. In the long term, patients will need ongoing care to manage reoccurrence of food cravings, particularly regarding high-sugar foods, fast food, and high-fat and high-carbohydrate foods, which are ubiquitous in today’s obesity-promoting environment.

### Supplementary Information

**Additional file 1: Table S1.** Internal consistency measures (standardized Cronbach’s alpha) for overall FCI and FCI subscales at each timepoint.

## Data Availability

The data that support the findings of this study are not openly available due to reasons of sensitivity and are available from the corresponding author upon reasonable request.
