# Supplementary material for: Food cravings after bariatric surgery: comparing laparoscopic sleeve gastrectomy and Roux-en-Y gastric bypass
Source: Eat Weight Disord. 2024 Jan 12;29(1):7. doi: 10.1007/s40519-023-01636-2 (PMC10786997; doi:10.1007/s40519-023-01636-2)
Supplement: Supplementary file 1 — Additional file 1: Table S1. Internal consistency measures (standardized Cronbach’s alpha) for overall FCI and FCI subscales at each timepoint. [file 40519_2023_1636_MOESM1_ESM.docx]

**Table S1.** Internal consistency measures (standardized Cronbach’s alpha) for overall FCI and FCI subscales at each timepoint.

| **Variable** | **Pre-Op** | **3-Months** | **6 Months** | **9 Months** | **12 Months** |
| --- | --- | --- | --- | --- | --- |
| **FCI All Component** | 0.91 | 0.90 | 0.89 | 0.87 | 0.91 |
| Sweets | 0.86 | 0.81 | 0.86 | 0.68 | 0.83 |
| Fast Food | 0.69 | 0.59 | 0.52 | 0.48 | 0.64 |
| High Fat | 0.81 | 0.76 | 0.76 | 0.83 | 0.76 |
| High Carb | 0.79 | 0.77 | 0.73 | 0.79 | 0.81 |

Abbreviations: FCI, food craving inventory.
